# Intraocular pressure reduction and neuroprotection conferred by bone marrow-derived mesenchymal stem cells in an animal model of glaucoma

**DOI:** 10.1186/s13287-015-0168-0

**Published:** 2015-09-16

**Authors:** Christophe Roubeix, David Godefroy, Céline Mias, Anaïs Sapienza, Luisa Riancho, Julie Degardin, Valérie Fradot, Ivana Ivkovic, Serge Picaud, Florian Sennlaub, Alexandre Denoyer, William Rostene, José Alain Sahel, Stéphane Melik Parsadaniantz, Françoise Brignole-Baudouin, Christophe Baudouin

**Affiliations:** INSERM, U968, Paris, F-75012 France; UPMC Université Paris 06, UMR_S 968, Institut de la Vision, Paris, F-75012 France; CNRS, UMR_7210, Paris, F-75012 France; Institut des Maladies Métaboliques et Cardiovasculaires, INSERM UMR 1048, 31432 Toulouse cedex 4, France, Toulouse, France; Centre Hospitalier National d’Ophtalmologie des Quinze-Vingts, INSERM-DHOS CIC 503, Paris, F-75012 France; University Paris Descartes, Sorbonne Paris Cité, Paris, F-75006 France; Faculté de Pharmacie de Paris, University Paris Descartes, Sorbonne Paris Cité, Paris, F-75006 France; Department of Ophthalmology, Hôpital Ambroise Pare, AP HP, Boulogne, F-92100 France; University Versailles St Quentin en Yvelines, Montigny-Le-Bretonneux, F-78180 France

## Abstract

**Introduction:**

Glaucoma is a sight-threatening retinal neuropathy associated with elevated intraocular pressure (IOP) due to degeneration and fibrosis of the trabecular meshwork (TM). Glaucoma medications aim to reduce IOP without targeting the specific TM pathology, Bone-marrow mesenchymal stem cells (MSCs) are used today in various clinical studies. Here, we investigated the potential of MSCs therapy in an glaucoma-like ocular hypertension (OHT) model and decipher in vitro the effects of MSCs on primary human trabecular meshwork cells.

**Methods:**

Ocular hypertension model was performed by cauterization of 3 episcleral veins (EVC) of Long-Evans male rat eyes. MSCs were isolated from rat bone marrow, amplified in vitro and tagged with quantum dot nanocrystals. Animals were distributed as 1) MSCs group receiving 5.10^5^cells/6μl Minimum Essential Medium and 2) MEM group receiving 6μl MEM (n = 10 each). Injections were performed into the anterior chamber of 20 days-hypertensive eyes and IOP was monitored twice a week for 4 weeks. At the end of experiment, cell distribution in the anterior segment was examined in confocal microscopy on flat mounted corneas. Moreover, we tested in vitro effects of MSCs conditioned medium (MSC-CM) on primary human trabecular meshwork cells (hTM cells) using Akt activation, myosin phosphorylation and TGF-β_2_-dependent profibrotic phenotype in hTM cells.

**Results:**

We demonstrated a rapid and long-lasting in vivo effect of MSCs transplantation that significantly reduced IOP in hypertensive eyes induced by EVC. MSCs were located to the ciliary processes and the TM. Enumeration of RGCs on whole flat-mounted retina highlighted a protective effect of MSCs on RGCs death. In vitro, MSC-CM promotes: (i) hTM cells survival by activating the antiapoptotic pathway, Akt, (ii) hTM cells relaxation as analyzed by the decrease in myosin phosphorylation and (iii) inhibition of TGF-β_2_-dependent profibrotic phenotype acquisition in hTM cells.

**Conclusions:**

MSCs injection in the ocular anterior chamber in a rat model of OHT provides neuroprotective effect in the glaucoma pathophysiology via TM protection. These results demonstrate that MSCs constitute promising tool for treating ocular hypertension and retinal cell degeneration.

**Electronic supplementary material:**

The online version of this article (doi:10.1186/s13287-015-0168-0) contains supplementary material, which is available to authorized users.

## Introduction

Glaucoma is a neurodegenerative disease affecting approximately 70 million people worldwide, and is the second leading cause of irreversible blindness. In contrast to other degenerative eye diseases, glaucoma may appear early in life, and consequently requires long-term ophthalmic medication. Primary open-angle glaucoma (POAG) is characterized by a progressive retinal ganglion cells (RGCs) death, which induces progressive loss in the visual field and acuity. Elevation of the ocular pressure, the major risk factor for RGCs death, occurs due to degeneration of the ocular tissue controlling aqueous humor outflow, namely the trabecular meshwork (TM). Indeed, TM degeneration in glaucoma is associated with the loss of TM cells, mainly through apoptotic mechanisms and oxidative stress, as well as extracellular matrix (ECM) remodeling and accumulation [1, 2]. It also results from changes in trabecular cell contractility associated with an increase in transforming growth factor beta 2 (TGF-β_2_) in the aqueous humor (AH) [3, 4]. These pathological changes lead to an increase in AH outflow resistance that raises intraocular pressure (IOP) and subsequently affects the entire neuronal visual pathway through RGCs degeneration mechanisms [5]. Current therapies for POAG aim to lower IOP by medication or surgery. However, these treatments do not specifically target the pathologic mechanisms involved in TM degeneration or in the RGCs apoptosis. As a result, these intricate mechanisms could explain some treatment failures in glaucoma. New therapeutic strategies that can encompass all facets of the disease—IOP-lowering medications as well as neuro-protective and TM-protective agents—are therefore still needed.

Mesenchymal stem cells (MSCs) are adult pluripotent stem cells that today have become an attractive new tool to treat various degenerative diseases. MSCs exist in all organs and tissues [6], and have been characterized by the expression of surface markers such as CD73, CD90, CD29, and CD105 [7] and their ability to differentiate in vitro into several cell types (i.e., osteoblasts, adipocytes, chondroblasts, and fibroblasts) [7]. The efficacy of MSCs therapy was originally attributed to the cells’ capacity to restore a population of differentiated cells through transdifferentiation [8]. However, MSCs also secrete a broad range of bioactive factors that constitute their secretome, which is responsible for the trophic (angiogenic, anti-apoptotic, and organ-intrinsic precursor or stem cell proliferation), immunomodulatory, anti-scarring, and chemoattractant effects of MSCs [9, 10]. In neurodegenerative diseases, MSCs secrete neurotrophic factors (such as brain-derived neurotrophic factor (BDNF), nerve growth factor (NGF), or vascular endothelial growth factor (VEGF)), directly promoting neural cell survival and cell growth or acting on the microenvironment of the neural tissue [11]. Recently, several preclinical studies using neurological disease models confirmed the high value of MSCs as sources of factors able to protect and promote tissue regeneration [12, 13]. In fact, cell-based therapies, such as gene-based therapies, offer powerful therapeutic tools, but their transfer to the clinical context remains difficult at times depending on the disease and the tissue involved. These new therapeutic means have found a convenient organ with the eye because it is directly accessible to therapeutic interventions, protected from the systemic circulation, and easy to examine so that drug efficacy and side effects can be monitored [14].

MSCs have also been proposed for the treatment of retinal diseases such as glaucoma [15], retinitis pigmentosa [16], and age-related macular degeneration [17]. Using a mouse ocular hypertension model, Manuguerra-Gagné et al. [18] reported that MSCs injection into the anterior chamber (AC) could induce TM regeneration and reactivate local neural progenitors in the ciliary body pigmented epithelium. However, this study did not examine the molecular mechanisms of TM protection and the effect on RGCs loss.

Here, we aimed to define the ability of bone marrow-derived MSCs to reduce IOP and protect the integrity of TM cells. Therefore, we first analyzed the impact of MSCs intracameral injection on lowering IOP in a preclinical model of ocular hypertension induced by episcleral vein cauterization (EVC). To decipher the mechanisms involved in the MSCs-dependent decrease in IOP, we performed in vitro experiments to assess the effect of MSCs with conditioned medium (MSC-CM) on primary human trabecular meshwork (hTM) cell properties in terms of viability, contractility, and expression of ECM compounds. This MSCs therapy may offer a new way to promote trabecular and RGCs protection to treat glaucoma, this severe sight-threatening disease.

## Methods

### Animals

Fifty male 8-week-old Long Evans rats weighing 300–350 g were purchased from Janvier Laboratories (Le Genest-Saint-Isle, France). Animals were kept in pathogen-free conditions with food and water available ad libitum and were housed in a 12-hour light/12-hour dark cycle. All experiments were performed after evaluation and approval by the Institutional Animal Care and Use Committee, Comité d'éthique pour l’expérimentation animale Charles Darwin (reference number: 03858.02), in accordance with the guidelines from Directive 2010/63/EU of the European Parliament on the protection of animals used for scientific purposes. All procedures were performed under anesthesia and all efforts were made to prevent any pain.

### EVC ocular hypertension model

A surgical model of ocular hypertension was induced in the right eye (RE) of each rat by cauterization of three episcleral veins after conjunctival dissection under general anesthesia—intraperitoneal injection of Ketamine 1000^®^ 100 mg/kg (Virbac, Vauvert, France) and Xylazine 10 mg/kg (Bayer HealthCare, Whippany, NJ, USA)—as reported elsewhere [19]. The left eye (LE) underwent conjunctival dissection only as a control. After surgery, IOP was monitored every 5 or 6 days using a handheld tonometer (TonoLab^®^; Medtronics, Jacksonville, FL, USA) without sedation. Animals presenting low or unstable IOP during a 21-day period after the surgery were excluded. Animals were treated 21 days after the surgery and IOP was monitored every 3–4 days for 24 other days in a blind manner.

### Enzyme-linked immunosorbent assay

The concentrations of TGF-β_2_ in the AC of cauterized eyes (REs) and contralateral eyes (LEs) were measured using enzyme-linked immunosorbent assay (ELISA) (DuoSet^®^ DY302; R&D Systems, Minneapolis, MN, USA). AHs were sampled from hypertensive eyes 21 days after surgery and pooled to obtain at least 50 μl per assay. Ninety-six-well plates were coated overnight with mouse anti-TGF-β_2_ capture antibody, and then nonspecific binding was blocked for 2 hours with blocking buffer. Samples of AH or serial dilutions of standards of rat recombinant TGF-β_2_ were incubated for 2 hours, washed, and then incubated with anti-TGF-β_2_ detection antibody for 2 hours, followed by a 20-minute incubation period with horseradish peroxidase-conjugated streptavidin. Reaction product was detected using color reagent. The color absorbance was read (450–570 nm, Infinite1000^®^; TECAN, Männedorf, Switzerland) and averaged from 10 measurements per well.

### MSC isolation and conditioned medium collection

Bone marrow was obtained from femur cavities of 8-week-old Long Evans rats (Janvier Laboratories) after flushing in minimal essential medium (αMEM), 10 % fetal calf serum (FCS), and 1 % penicillin/streptomycin (PS) (Invitrogen, Carlsbad, CA, USA). Cells were incubated in 75 cm^2^ flasks (200,000 cells/cm^2^) at 37 °C in 5 % CO_2_ humidified air. Nonadherent cells were discarded after 72 hours and MSCs were then routinely cultured until their use at passage 4 and characterized as shown in Additional file 1. MSCs were maintained in culture for a longer period until losing the CD73 specific marker as controlled by flow cytometry (passage 7, data not shown), defining differentiated MSCs (dMSCs).

At near confluence, the cell monolayer was washed before adding either serum-free Dulbecco’s modified Eagle’s medium (DMEM) + 1 % PS or serum-free Neurobasal^®^ medium containing 2 mM l-glutamine, 1 % gentamycin (Invitrogen) for 24 hours. Supernatant was collected, centrifuged at 1200 rpm for 2 minutes, and stored at −80 °C until further use for experiments on hTM (CliniSciences, Nanterre, France) and primary RGCs.

### MSC injection and tracking system

Before injection, MSCs were washed with phosphate buffer solution (PBS), trypsinized, and labeled with Quantum Dot (QD) Fluorescent Nanocrystals (Qtracker^®^ 655 Cell Labeling kit; Invitrogen). Cells were suspended in αMEM for each injection (5 × 10^5^ MSCs/6 μl). Cell suspension was checked under fluorescent microscopy for QD integration into MSCs.

MSCs were injected into the AC 21 days after EVC. IOP was measured every 3–4 days for 24 days in both eyes of each animal randomly distributed into four groups: hypertensive eyes injected with 6 μl MEM (EVC + MEM; *n* = 10); hypertensive eyes injected with 6 μl of 5 × 10^5^ MSCs suspension (EVC + MSCs; *n* = 12); hypertensive eyes injected with 6 μl of 5 × 10^5^ dMSCs suspension (EVC + MSCs; *n* = 5); and normotensive eyes injected with 6 μl of 5 × 10^5^ MSC suspension (MSCs; *n* = 5).

During injection, animals were maintained under general anesthesia using isoflurane inhalation (induction 5 %, then 2 %). The injection was performed through a glass capillary (TransferTip^®^; Eppendorf, Hauppauge, NY, USA)) under a binocular surgical microscope (Leica F18; Leica Microsystems, Nanterre, France).

### Immunostaining

At the end of the experiments, the animals were euthanized and the eyes were immediately removed, fixed in 4 % paraformaldehyde for whole flat-mounted cornea and retina microdissections, or embedded in an optimal cutting-temperature compound for snap-freezing (OCT, Tissue-Tek; Bayer Diagnostic, Puteaux, France). Retinas were incubated for 2 hours in a blocking-permeabilizing solution of PBS containing 10 % bovine serum albumin (BSA) (Vector Labs, Burlington, ON, Canada), 2 % Triton and 0.5 % Tween20 (Sigma-Aldrich, St. Louis, MO, USA). Samples were incubated for 3 days at 4 °C with unlabeled mouse anti-Brn3a 1/100 (Merck, Darmstadt, Germany), washed with PBS six times for 20 minutes, and mounted in Fluoromount™ (Sigma-Aldrich).

To visualize QD-labeled MSCs using fluorescent microscopy, flat-mounted corneas and eye cryosections (12 μm thick) were incubated in the blocking-permeabilizing solution (10 % BSA, 2 % Triton, and 0.5 % Tween20) before actin fibers and nuclei staining with Phalloidin (Life Technologies, Carlsbad, CA, USA) and 4′,6′-diamidino-2-phénylindole (DAPI) (Vectashield; Vector Labs, Burlingame, CA, USA) respectively. Whole flat-mounted retina and cornea were scanned with the Hamamatsu Nanozoomer Digital Pathology (Hamamatsu Photonics, Tokyo, Japan). Magnifications were performed with a laser-scanning confocal microscope (FV1000; Olympus, Philadelphia, PA, USA). Acquisitions were conducted using the Olympus Fluoview software version 4.1.

### Western blot analysis

Cell monolayers were homogenized in lysis buffer (25 mM Tris pH 7.5, 150 mM NaCl, 0.5 % sodium deoxycholate, 1 % Triton, 0.1 % SDS) with a protease and phosphatase inhibitor cocktail (Sigma-Aldrich). Cell homogenates were centrifuged (10,000 rpm, 10 minutes, 4 °C). Protein concentrations were measured using Bradford reagent (Sigma-Aldrich). Equal amounts of total protein were denatured for 10 minutes at 95 °C (5 μg) with NuPage reducing agent (Invitrogen) before electrophoresis in 4–12 % Tris-Glycine gel (Novex; Invitrogen); the membranes were transferred to nitrocellulose, blocked with TBS–Tween 0.1 % + Blotting Grade blocker 5 % (Bio-Rad, Hercules, CA, USA) at room temperature for 2 hours, and probed overnight at 4 °C with antibodies against β-actin (1:10,000; Sigma-Aldrich), phospho-Akt (p-Akt) (1:500; Cell Signaling, Danvers, MA, USA) and phospho-myosin (p-MLC) (1:500; Cell Signaling). The membranes were washed three times with TBS–Tween 0.1 % and incubated for 30 minutes with the appropriate peroxidase-conjugated secondary antibody (1:10,000). Cells were detected using an enhanced chemiluminescence reaction using ECL Plus detection reagents (GE Healthcare, Orsay, France). Protein bands were quantified by densitometry using ImageJ software (NIH, Bethesda, MD, USA) and the results expressed as the protein of interest/β-actin ratio.

### Real-time PCR

Total mRNA from cell monolayers was extracted using the NucleoSpin RNA II extraction kit (Macherey-Nagel, Düren, Germany). RNA content was measured using a NanoDrop detector (ND-1000 spectrophotometer; Wilmington, DE, USA); cDNA was synthesized from equal amounts of RNA (800 ng) using Multiscribe reverse transcriptase (TaqMan Reverse Transcription Reagents; Applied Biosystems, Life Technologies, Carlsbad, CA, USA). Sample concentrations were adjusted to 5 ng/μl cDNA. The reaction mixture containing 25 ng cDNA per well was preheated at 95 °C for 10 minutes, followed by 40 cycles (95 °C for 15 seconds and 60 °C for 1 minute). Each assay, carried out in triplicate, was normalized by amplifying the housekeeping cDNA hypoxanthine guanine phosphoribosyltransferase (HPRT) (ID Hs02800695;). Target cDNA was amplified using the 7300 Real-Time PCR system (Applied Biosystems, Foster City, CA, USA) with assays-on-demand primers for human alpha-smooth muscle actin (α-SMA) (ID Hs00426835m1), type III collagen (ID Hs00943809), and type IV collagen (ID Hs00266237; Applied Biosystems). Relative quantification of target mRNA was assessed according to the comparative Ct method (2^–∆∆Ct^); results are presented as the relative fold change compared with unstimulated control.

### Human trabecular cell culture and treatment

This study used commercial hTM primary cells (ref. 6590-SC; CliniSciences) and did not use any material taken from patients, so no consent was needed.

hTM cells were obtained from 24-year-old adult donors. Cells were maintained in culture at 37 °C in an atmosphere of 95 % air and 5 % CO_2_ in DMEM + 10 % heat-inactivated (56 °C, 60 minutes) FCS + 1 % PS.

After trypsinization, 10^5^ hTM cells were seeded in six-well plates and tested in different conditions: serum-free DMEM (Control), 20 ng/ml exogenous TGF-β_2_ (TGF-β_2_), MSC-CM, or association of TGF-β_2_ (20 ng/ml) and MSC-CM (TGF-β_2_ + MSC-CM). After washing, proteins were recovered 3 hours after treatment, whereas RNA was recovered 72 hours after treatment, with the adapted buffer, as described previously.

### hTM luminescent viability assay

After trypsinization, 10^5^ hTM cells were seeded in 96-well plates. Cells were exposed 24 hours later to an increasing concentration of benzalkonium chloride (BAC) (Santen Pharmaceutical) from 1.5 × 10^−4^ % (0.5 μM) to 5 × 10^−4^ % (1.5 μM) for 24 hours. In each well, half the volume of culture medium was removed and replaced by CellTiter-Glo^®^ reagent (CTG) (Promega, Madison, WI, USA). Ten minutes after adding CTG, the luminescent signal was measured by spectrophotometry (Infinite1000^®^; TECAN, Männedorf, Switzerland) to quantify the adenosine triphosphate (ATP) content as an average from nine measurements per well.

### Quantification of RGCs in whole flat-mounted retina

Eight microscopic images were captured using a 20× objective of the peripheral and central area in whole flat-mounted retina labeled with brain-specific homeobox/POU domain protein 3A (Brn3a) antibody with a Zeiss fluorescence microscope (Carl Zeiss SpA, Arese, Milan, Italy) equipped with a digital camera (Axio Cam HRC; Carl Zeiss) and image acquisition software (AxioVision; Carl Zeiss). Automatic quantification of RGC nuclei was assessed in a blind manner with MetaMorph software (Universal Imaging, West Chester, PA, USA).

### Statistical analysis

Values are expressed as mean ± standard error of the mean. ELISA, western blot (WB), and quantitative RT-PCR data were analyzed using the one-way or two-way analysis of variance test followed by the post-hoc test and nonparametric Mann–Whitney *U*-test. Statistical analyses were performed using GraphPad Prism version 6.03 (GraphPad, San Diego, CA, USA). All statistical tests were two-sided and performed at the significance level of *p* <0.05.

## Results

### Ocular hypertension model validation: effects on IOP and AH TGF-β_2_ levels

**Fig. 1:**
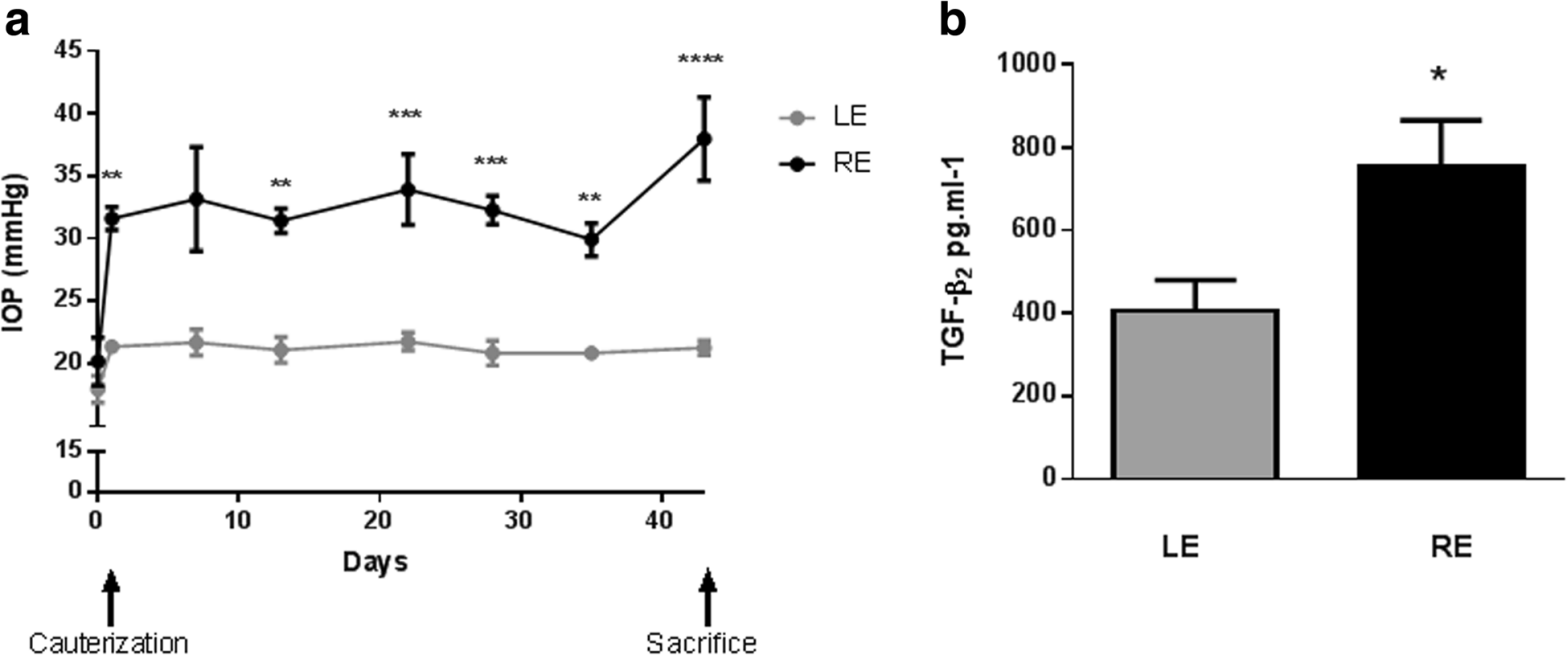
Effect of EVC on IOP and AH TGF-β_2_ expression. Intraocular pressure (*IOP*) monitoring of right eyes (*REs*) exposed to three EVCs (*black bar*) and of control left eyes (*LEs*; *gray bar*) over 44 days **a**. AH levels of transforming growth factor beta 2 (*TGF-β*_*2*_) of both cauterized (RE) and control (LE) eyes sampled 21 days after EVC **b**. *p <0.05, **p <0.01, ***p <0.001, ****p <0.0001 vs. control

An increase in the IOP levels is the main risk factor for the development of glaucoma [20] and is correlated with an increase of TGF-β_2_ in the AH [20]. We therefore examined whether our animal model of glaucoma was associated with an increase in AH TGF-β_2_ as the IOP increased_._ Cauterization of three episcleral veins induced a decrease in AH outflow and consequently an increase in IOP. Twenty-four hours after surgery, the mean IOP elevation in the cauterized eyes (REs) versus the contralateral eye (LE) was 8.5 ± 2.8 mmHg (*n* = 5; *p* <0.001) (Fig. 1a). IOP remained stable for approximately 6 weeks with an overall mean increase of 10.4 ± 2.0 mmHg in the RE vs. LE over this period of time (Fig. 1a). Moreover, the AH concentration of TGF-β_2_ showed a significant twofold increase in the RE compared with the LE (*p* <0.05) (Fig. 1b).

### MSCs transplantation decreases IOP in a rat model of ocular hypertension

**Fig. 2:**
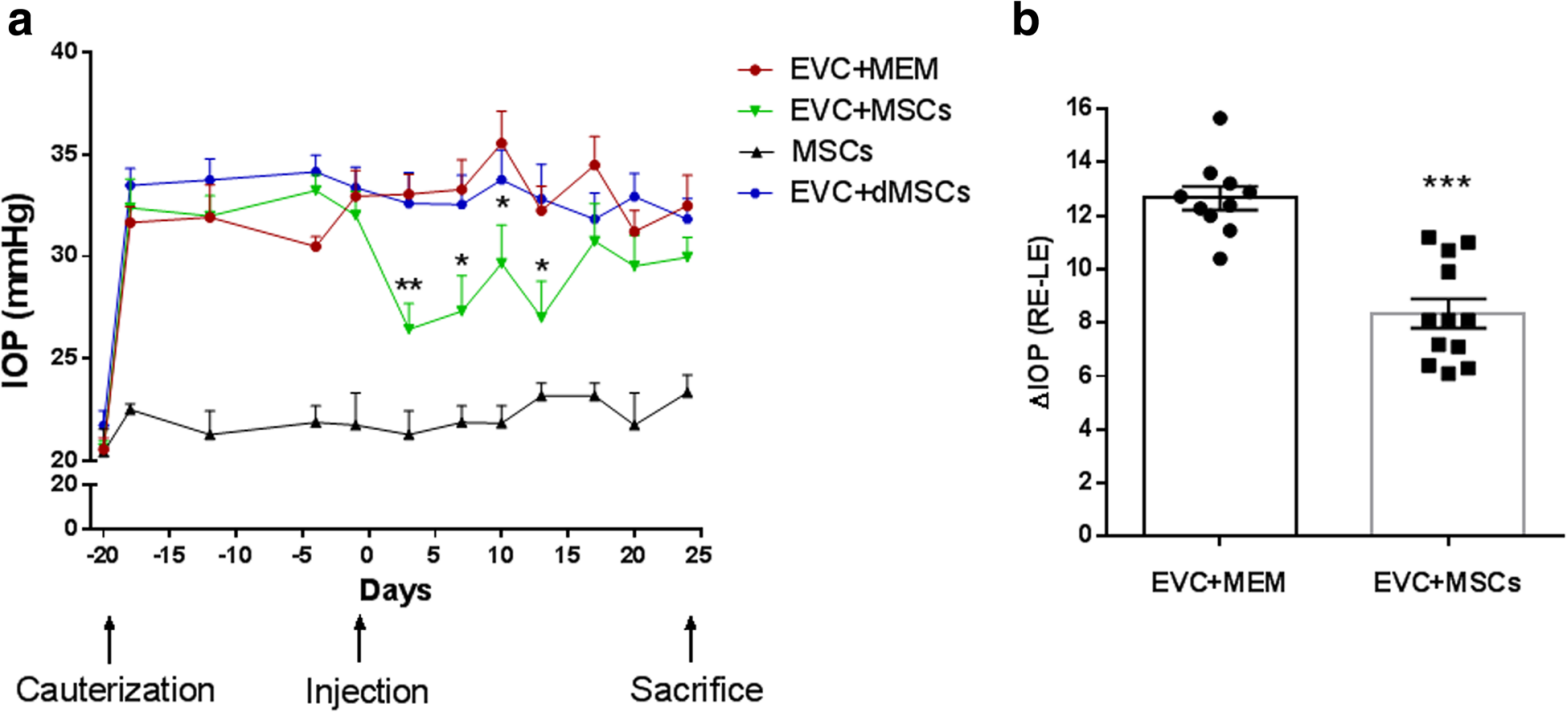
MSC transplantation decreases IOP in a rat model of ocular hypertension. The intraocular pressure (*IOP*) was monitored in both cauterized (right eye (*RE*)) and noncauterized (left eye (*LE*)) eyes. IOP measurements of the RE were reported for each animal per group: hypertensive eyes injected with 5 × 10^5^ MSCs (EVC + MSCs, *green bar*; *n* = 12), hypertensive eyes injected with culture medium alone (EVC + MEM, *red bar*; *n* = 10), or normotensive eyes injected with 5 × 10^5^ MSCs (MSCs, *black bar*; *n* = 5) **a**. Individual average of delta IOP (ΔIOP = RE IOP − LE IOP) measurements from injection to sacrifice times were calculated and compared between the EVC + MSCs and EVC + MEM groups **b** *f*p* <0.05, **p <0.05, ***p <0.001 vs. EVC + MEM. *dMSCs* differentiated mesenchymal stem cells, *EVC* episcleral vein cauterization, *MEM* minimum essential medium, *MSCs* mesenchymal stem cells

To assess the therapeutic potential of MSCs in glaucoma, we evaluated the intracameral injection of MSCs in our model. Two days after the MSCs injection, the IOP level in the EVC + MSCs group decreased by 5.1 mmHg, showing a significant difference compared with the glaucomatous rats injected with a MEM control solution (EVC + MEM group) (*n* = 10; *p <*0.01). The MSCs-induced decrease in IOP remained significantly different from the EVC + MEM group for 13 days (Fig. 2a) (*n* = 10; *p* <0.05 and *p* <0.01). To examine whether this IOP decrease was specific to the glaucomatous condition, we also injected MSCs into control rats having a normal ocular pressure (MSC group). The IOP monitoring after the injection did not show any modification in IOP during the experiment in this group (Fig. 2a). Furthermore, to ensure that the injection of biological material could participate in the IOP decrease, 5 × 10^5^ dMSCs (EVC + dMSCs) were injected into hypertensive eyes. No IOP modification was observed (Fig. 2a), confirming the specific effect of nondifferentiated MSCs on the IOP reduction.

The average of seven measurements of delta IOP (difference between cauterized RE and noncauterized LE), from the time of injection to the day of sacrifice for each animal, was significantly lower in the EVC + MSCs group than in the EVC + MEM group during this period.

### Tracking MSCs in the AC after EVC

**Fig. 3:**
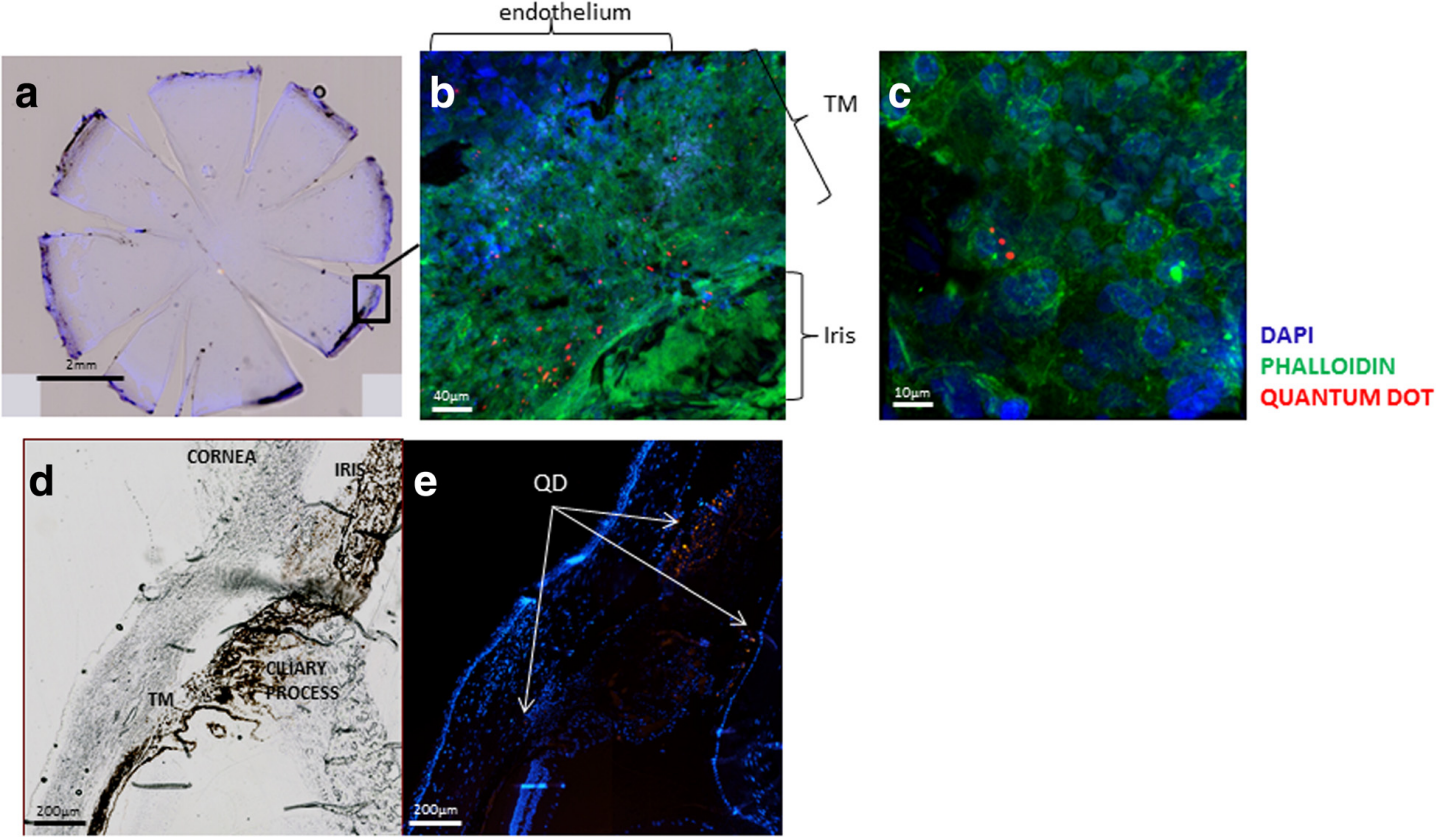
Tracking of MSCs after EVC in the AC. Whole flat-mounted cornea viewed with a digital fluorescence scanner (Nanozoomer) **a**. Confocal microscopy images at two different magnifications (**b** ×200, **c** ×800) of flat-mounted cornea showing cell nuclei (DAPI; *blue*), actin fibers (Phalloidin; *green*) and quantum dot (QD)-labeled MSCs (*red*) injected 21 days after EVC and found 23 days later at the time of sacrifice. Representative images of AC angle with contrast phase **d** and fluorescence microscopy **e** showing cell nuclei (DAPI; *blue*) and QD-labeled MSCs on the cornea endothelium, ciliary processes, and trabecular meshwork (*TM*) on a cryostat section (*red*). *DAPI* 4′,6′-diamidino-2-phénylindole

To investigate the origin of the IOP decrease induced by MSCs injection, we examined the ocular distribution of the MSCs after injection, in particular to determine whether they were able to migrate into the TM and to survive. For long-term tracing of the MSCs fate, we used QD Fluorescent Nanocrystal labeling. Six rats were injected with the labeled MSCs (5 × 10^5^ MSCs). After fixation of the tissues, the distribution of MSCs in the AC was investigated in whole flat-mounted corneas. Twenty-four days after the injection of 5 × 10^5^ MSCs, cells were found located near the iridocorneal angle, on the corneal endothelium, and in the TM (Fig. 3a–c). The higher magnification confirmed the intracellular integration of QD (Fig. 3c, ×800). Another set of six injected rats were used for cryosectioning (Fig. 3d, e). As observed on flat-mounted corneas, QD-labeled MSCs were found on the corneal endothelium, on the iris, and in the TM but also in the ciliary processes (Fig. 3e).

### MSCs transplantation improves in vivo peripheral RGCs survival

**Fig. 4:**
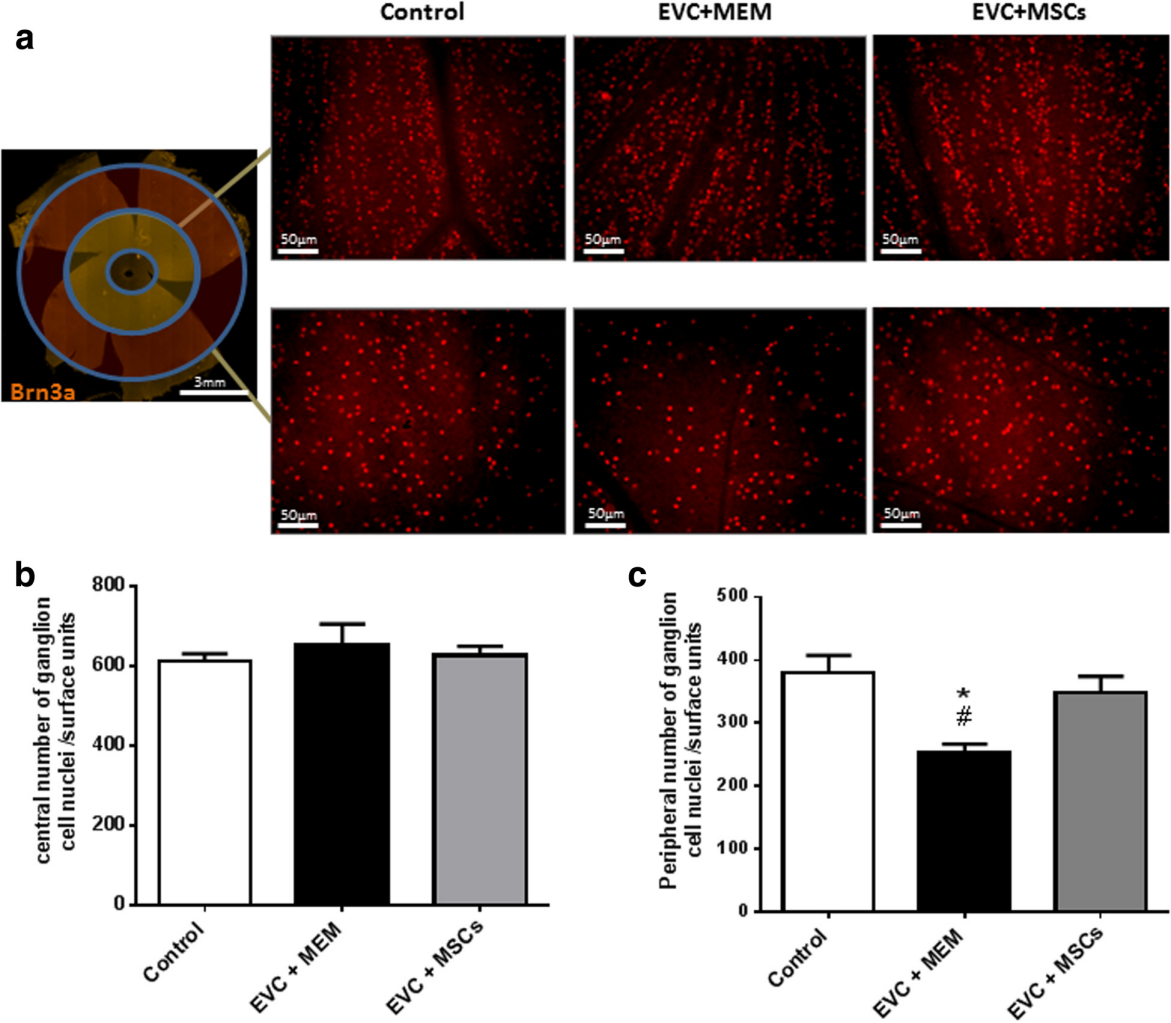
MSCs transplantation improves RGCs survival. Whole flat-mounted retina immunolabeled with brain-specific homeobox/POU domain protein 3A (*Brn3a*) antibody. Representative images (×200) of the peripheral or central area showing immunopositive staining of RGCs in noncauterized (Control) and cauterized eyes injected with MEM (EVC + MEM) or MSCs (EVC + MSCs) **a** Quantification of central **b** and peripheral **c** RGC densities per retina using automated nuclei counting in the control group (*n* = 5), EVC + MEM group (*n* = 5), and EVC + MSCs group (*n* = 5). The average RGCs density was obtained from eight peripheral and central images per retina and per area. Data expressed as mean ± standard error of the mean. *p <0.05 vs. control, #*p* <0.05 vs. EVC + MSCs. *EVC* episcleral vein cauterization, *MEM* minimum essential medium, *MSCs* mesenchymal stem cells

After confirmation of the beneficial effects of MSCs on the IOP and their incorporation into the anterior structures of the eye, we investigated the effect of intracameral MSC injection on RGCs degeneration. RGCs were immunostained with Brn3a antibody in whole flat-mounted retina. Images of the peripheral and central areas of the retina were taken (Fig. 4a). No significant effect was observed in the central retina on RGCs density (Fig. 4b). However, the EVC + MEM group exhibited a significant 33 % decrease (*p* = 0.016) in RGCs density in the peripheral retina compared with the control group. Interestingly, the RGCs density in the peripheral retina of the EVC + MSCs group was significantly higher as compared with the EVC + MEM group (*p* = 0.029) (Fig. 4c). The density of RGCs in the peripheral retina of hypertensive eyes treated with 5 × 10^5^ MSCs was not significantly different from the control normotensive noncauterized group (LEs, *p* = 0.40)*.* MSCs transplantation in the AC thus appeared to protect from the peripheral RGCs degeneration in the EVC hypertension model.

### MSC-CM induces TM primary cell survival and contractibility

To further understand the IOP decrease following MSCs injection, we investigated in vitro the impact of MSC-CM on three major mechanisms described to be involved in IOP regulation: TM cell viability, contractility, and TM cell phenotype transition*.*

**Fig. 5:**
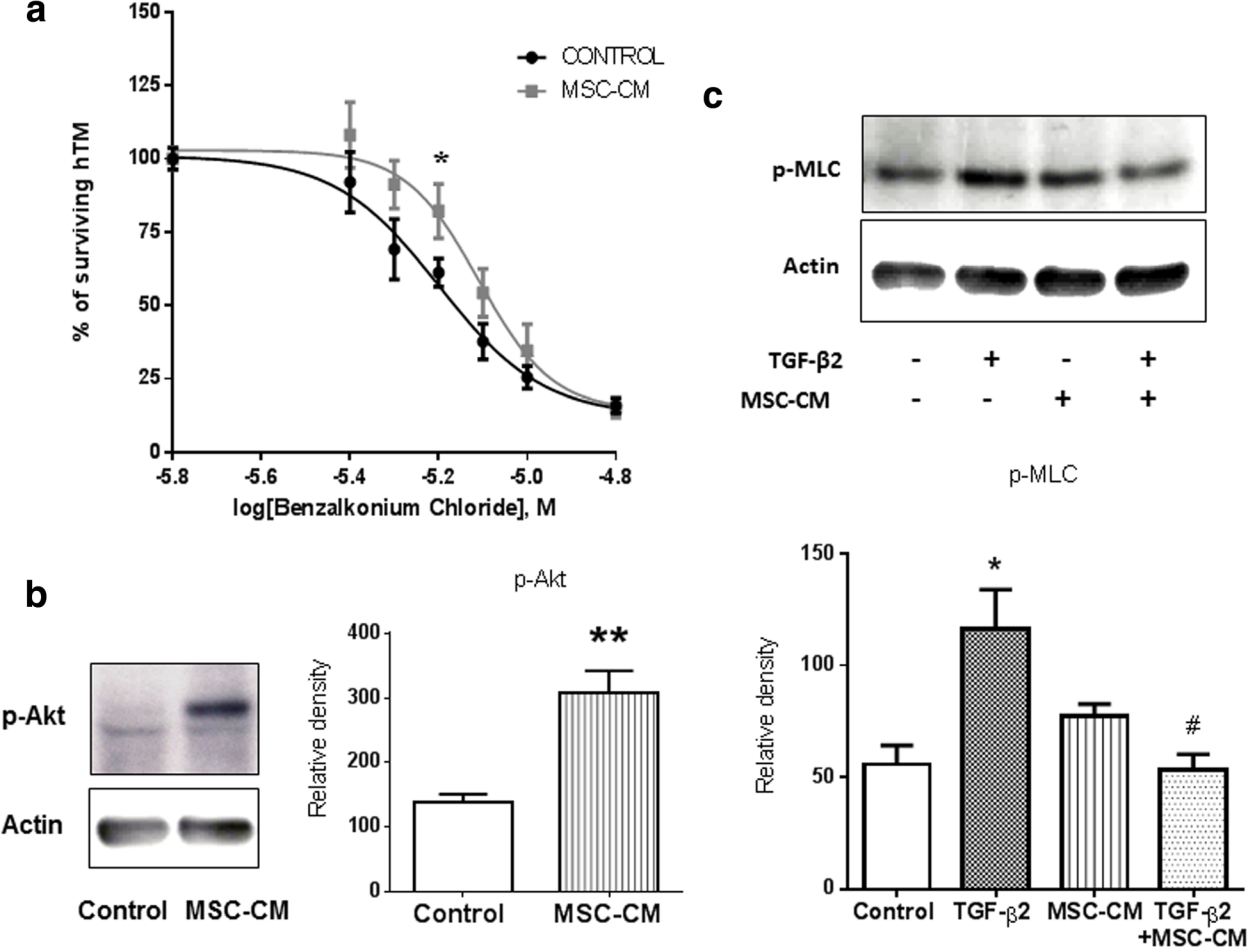
MSC-CM induces TM primary cell survival and contractility. Effect of MSC-CM on human trabecular meshwork (*hTM*) cell survival and contractility with or without transforming growth factor beta 2 (*TGF-β*_*2*_). hTM cell viability assessed by ATP quantification following 24 hours of increasing BAC concentration exposure with or without MSC-CM **a**. Immunoblot of phospho-Akt (*p-Akt*), ± MSC-CM, and phospho-myosin (*p-MLC*), ± MSC-CM and ± TGF-β_2,_ protein expression using WB in hTM cells was performed **b**, **c**. The relative density of protein bands were quantified, and the ratios of phosphorylated protein to actin were calculated (*n* = 4) **b**, **c**. *p <0.05 and ***p <0.001 vs. control culture condition, #*p* <0.01 vs. TGF-β_2_ condition. *MSC-CM* conditioned medium of mesenchymal stem cells

A previous study has shown a significant increase of TM cell apoptosis in the hypertensive eyes induced by EVC [19]. Thus, we first evaluated the effect of MSC-CM on hTM cell viability using an in vitro cytotoxic model. Cells are exposed for 24 hours to the cytotoxic effects of BAC, which was found to induce TM cell death in a dose-dependent manner. When co-applying MSC-CM, we observed a significant shift in the lethal concentration 50 (LC50) toxicity of BAC. LC50 was 6.3 × 10^−6^ M for hTM cells alone instead of 7.8 × 10^−6^ M for hTM cells maintained in MSC-CM (Fig. 5a). To confirm the beneficial effect of MSC-CM on hTM cell viability, we examined the activation by WB of a transduction factor involved in survival mechanisms of Akt on hTM cells exposed to the MSC-CM for 3 hours. We observed that hTM cells displayed a low level of Akt phosphorylation (p-Akt) in control, whereas MSC-CM revealed a major increase in p-Akt corroborating the protective effect observed in hTM cells exposed to the cytotoxic effect of BAC (Fig. 5b).

TM cell contractility is associated with a resistance in AH outflow. This mechanism involves TGF-β_2,_ known to be correlated with the IOP levels in AH of glaucoma patients [13, 14] and described to trigger the phosphorylation of the myosin fibers (MLC) inducing TM cell contraction [21], reduction of HA outflow, and then IOP increase. We exposed hTM cells to exogenous TGF-β_2_ (20 ng/ml) for 3 hours in standard culture medium ± MSC-CM. WB assays were performed to detect and quantify MLC phosphorylation (Fig. 5c). In this experimental condition, TGF-β_2_ triggered a twofold increase in p-MLC (Fig. 5c). Interestingly, MSC-CM strongly inhibited the effect of TGF-β_2_ on p-MLC induction (Fig. 5c). In fact, hTM cells exposed simultaneously to TGF-β_2_ and MSC-CM showed a significantly lower level of p-MLC than cells exposed to TGF-β_2_ alone.

For both markers, a beneficial effect was observed when hTM cells were exposed to MSC-CM. Quantification of the p-MLC and p-Akt bands were presented in histograms (*n* = 4).

### MSC-CM reduces trabecular mesenchymal transition in primary trabecular cells

**Fig. 6:**
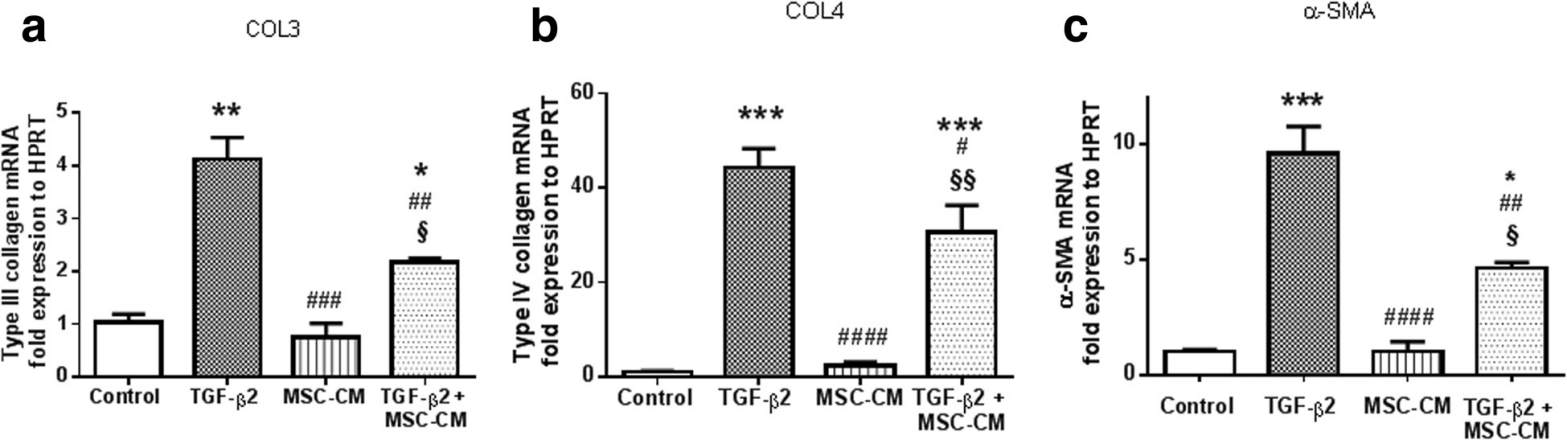
MSC-CM reduces epithelial mesenchymal transition-like phenomenon in primary trabecular cells. Effect of MSC-CM on transforming growth factor beta 2 (*TGF-β2*) induced collagen-3 (*COL-3*) **a**, collagen-4 (*COL-4*) **b**, and alpha-smooth muscle actin (α-SMA) **c** mRNA modification on hTM. The results are normalized to reference hypoxanthine guanine phosphoribosyltransferase (*HPRT*) genes. *p <0.01, **p <0.001, and ***p <0.0001 vs. control culture condition; #*p* <0.05, ##*p* <0.01, ###*p* <0.001, and ####*p* <0.0001 vs. TGF-β_2_ condition; §*p* <0.01 and §§*p* <0.0001 vs. MSC-CM condition (*n* = 5). *MSC-CM* conditioned medium of mesenchymal stem cells

The third mechanism of TM degeneration implies TM cell phenotype transition. TGF-β_2_ is known to induce phenotype transition in different cell types (i.e., epithelial, endothelial, or fibroblastic). This mechanism is responsible for the initiation of tissue fibrosis even in TM under glaucomatous conditions [22]. Therefore, we investigated the potential protective effect of MSCs on this trabecular mesenchymal transition (TMT) through the analysis of genetic expression of two ECM compounds, collagen-3 and collagen-4, and through a myofibroblast marker, α-SMA. First, we confirmed that hTM cells exposed to TGF-β_2_ for 72 hours acquired an increased mRNA expression of collagen-3 (Fig. 6a), collagen-4 (Fig. 6b), and α-SMA (Fig. 6c). Then we showed that this phenomenon was significantly reduced when cells were simultaneously exposed to TGF-β_2_ and MSC-CM. MSC-CM alone did not modify mRNA levels (Fig. 6a–c). MSC-CM inhibited the TGF-β_2_-dependent phenotype transition in hTM cells.

## Discussion

Since their identification by Friedenstein in the 1970s [23], interest in bone marrow-derived MSCs in various clinical contexts such as ischemic stroke [24], myocardial infarction [9, 25], and Crohn’s disease [26] has grown continuously. In the field of ocular diseases, bone marrow-derived MSCs have successfully been proposed to promote corneal healing [27–29], reduce dry eye in chronic graft-versus-host disease [30], to prevent photoreceptor degeneration [16, 17, 31], and also in glaucoma to protect the RGCs from degeneration after intravitreal injection [15, 32–35]. In this study, we originally show that MSCs transplantation into the anterior segment of the eye induces RGCs protection, through TM function and integrity improvement and IOP reduction.

Cell-based therapeutic strategies for TM regeneration have already been developed using induced pluripotent stem cells or MSCs. Abu-Hassan et al. [36] showed in an ex vivo perfused human AC model that induced pluripotent stem cells are both able to repopulate the degenerative TM and to acquire a TM cell morphology and phenotype to finally restore the TM filtrating property. The same conclusions have been reached with MSCs derived from TM injected into a mouse AC. Bone marrow-derived MSCs have also shown interesting properties for TM regeneration. Manuguerra-Gagné et al. [18] demonstrated a restoration of TM function after MSCs injection into a mice model of laser-induced hypertensive eyes. However, they demonstrated that MSCs mainly acted though their paracrine activity stimulating the resident TM stem cells. In fact, bone marrow-derived MSCs did not acquire TM cell phenotype in coculture with TM cells. Consequently, the interest of MSCs to fight TM degeneration seems to come essentially from the factors they secrete known to be involved in tissue homeostasis. Therefore, in this study we focused on bone marrow-derived MSC secretome potential to inhibit or alleviate TM degeneration and to restore a filtering function.

Despite the description of many glaucoma models in the literature (laser trabecular destruction, steroid-induced ocular hypertension, intracameral injection of latex microspheres, or autologous fixed red blood cells), there is still a need for a more accurate glaucoma model. Because is it difficult to reproduce lifelong progressive disease in animals, the EVC model seems to be relevant to study glaucoma therapeutic strategies. Many studies have characterized and used the EVC model, which matched or gathered many specific glaucoma features—not only an IOP increase with a reduction of AH outflow without affecting directly the TM, but also progressive RGCs degeneration, an AH TGF-β_2_ level increase, and TM cell death as observed in glaucomatous patients [3, 4]. Using this EVC hypertension model, we showed that a single injection of MSCs into the anterior segment of hypertensive rat eyes is able to rapidly and significantly reduce the increase in IOP induced by EVC. These results are in agreement with Manuguerra-Gagné et al. [18] in another preclinical ocular hypertension model in mice induced by 180° TM laser photocoagulation. Manuguerra-Gagné et al. demonstrated recently that MSCs transplantation into laser-induced hypertensive eyes of mice progressively reduces IOP and attains a maximal effect at 10 days post injection until the end of their 25-day study. In our study, the IOP lowering was seen as soon as 3 days after injection and was maintained for 13 days. This difference could be attributed to the different ocular hypertension animal models used. Indeed, in the laser model half of the entire TM was destroyed, compared with the EVC model in which the TM was not directly damaged and the TM injury was progressive and secondary to the increase in IOP [19].

Twenty-three days after QD-labeled MSCs transplantation, we demonstrated that MSCs were distributed within the AC, especially inside and around the TM. These results differ from those reported in Manuguerra-Gagné et al.’s [18] study, in which MSCs were no longer detected in the AC 96 hours post injection. These authors hypothesized that the injected MSCs escaped through the TM or died following their transplantation. QD staining is a more adapted system for tracking this type of cell, because it is able to show that MSCs remained integrated in the different parts of the anterior segment of the eye for a long period (at least 3 weeks) after injection into the AC.

While we demonstrated that MSCs were still present in the tissue after 3 weeks, the reduction in IOP induced by MSCs injection in the AC only lasted for approximately 2 weeks. We presumed that this transient MSCs effect results in either tissue resistance to MSC-CM or that the MSCs lose their ability to restore their tissue-filtrating property. It is known that the effect of MSCs is due to various molecules they synthesize and release. Furthermore, they progressively differentiate into the tissue type in which they are incorporated. Thus, we hypothesized that injected MSCs progressively transdifferentiate into specialized cells, which modify the nature of their secretome and finally lose their positive effect on the TM. The lack of effect on IOP observed with the injection of dMSCs in the model (passage 7 in vitro loss of CD73 expression; data not shown) leads us to think that the IOP reduction depends on MSCs pluripotent state.

Mechanisms involved in TM degeneration are still poorly known. The size of this tissue does not help investigation of the in vivo features. However, studies have highlighted major mechanisms involved in the loss of TM function, especially a fibrotic process including TM cell apoptosis and ECM remodeling and a defect in contractility/relaxation of TM cells. The defects lead progressively to IOP increase. In an original manner, using in vitro approaches, our work investigated whether MSCs display valuable properties for these hallmarks.

TM cells are known to acquire contractile properties in response to pharmacological stimulations. Specifically, TM cell contraction has been reported to reduce AH outflow, whereas TM cell relaxation increased AH outflow facility and thus decreased IOP [37, 38]. Using a cellular TGF-β_2_-induced model of glaucoma, we showed for the first time that MSC-CM prevented the TGF-β_2_-dependent phosphorylation of myosin fibers on hTM cells. The Akt pathway activation (a serine/threonine kinase intracellular pathway involved in cell survival) triggered by MSC-CM has already been observed in different kind of cells, like endothelial cells [39], fibroblasts [40], or neurons [41]. We showed, first, that BAC exhibits a cytotoxic effect on hTM cells at low concentration (2 × 10^−4^ %; 0.7 μM); that is, approximately 100 times less than in common glaucoma eye drops (2 × 10^−2^ %; 70 μM). Secondly, we demonstrated that MSC-CM induced Akt phosphorylation on hTM cells, promoting efficiently their survival in a BAC-induced cytotoxic in vitro model.

Growing interest is also focused on the TMT-like phenomenon concerning trabecular cells [22]. Indeed, this mechanism, occurring in chronic inflammatory disease, is defined as initiating fibrosis in different tissues. Here, we confirmed in human primary trabecular cells that exogenous TGF-β_2_ was able to induce TMT through the increase of ECM compound mRNA such as collagen-3, collagen-4, and the myofibroblast marker α-SMA. As previously shown in other fibrosis models in various tissues [42, 43], we also observed that MSC-CM significantly reduced collagen-3 and collagen-4. Moreover, the increase in α-SMA mRNA induced by TGF-β_2_ confirmed that MSC-CM could be useful to block TM fibrosis. Thus, we showed the direct impact of MSC-CM in vitro on TM cell survival, contractility, and the reduction of the profibrotic phenotype. These MSC-CM specific effects on TM cells in vitro could explain the rapid IOP decrease, even if transient, that occured after MSCs intracameral injection.

In glaucoma, the loss of vision is related to the degeneration of RGCs. RGCs protection after MSCs intravitreal injection has been evidenced in different animal models of retina degeneration such as optic nerve crush [44] or optic tract transection [45] and ocular hypertension induced by photocoagulation of the TM [15]. More recently, the same conclusion has been reached using organotypic retina explant cultures [34]. In an in vitro study, we could observe a direct trophic effect on a purified primary rat RGCs culture using a cell viability assay (data not shown). Together, these studies showed that MSCs played favorable effects either directly on RGCs survival and outgrowth or indirectly by acting on the RGCs microenvironment. Direct neuroprotective and/or neuroregenerative therapeutic strategies constitute a major challenge but, until today, they have not brought any relevant clinical effects. Because ocular hypertension is the most important risk factor for RGCs death in glaucoma, we have innovatively demonstrated in this work that MSCs injection into the AC of hypertensive eyes is able to greatly reduce IOP resulting in an efficient protection of RGCs from degeneration processes; this latter effect of IOP reduction protecting RGCs, as already demonstrated by others [19]. In fact, the hypertensive eyes injected with MSCs (EVC + MSCs) did not develop the significant loss of peripheral RGCs (8 %, *p* = 0.40) that was observed in the EVC + MEM group (33 %, *p* = 0.016). RGCs density in the peripheral retina was significantly higher in MSCs-injected hypertensive eyes. Peripheral RGCs are known to be more sensitive than central RGCs to high IOP, as shown by the retinal damage characterization in the ocular hypertension model [20, 46]. We suggest that the neuroprotective effect observed here could be mainly due to the IOP decrease that occurs after MSCs injection.

## Conclusion

The rationale behind using MSCs in glaucoma is based on pathophysiologic clues. First, the increase in TGF-β_2_ levels in the AH of glaucoma patients induces phenotype changes through trabecular to mesenchymal transition resulting in fibrotic-like mechanisms involving cell apoptosis and ECM remodeling. Further, the peripheral RGCs death leading to progressive vision loss justifies the potential therapeutic use of MSCs. MSCs therapy is already used today, with promising therapeutic results, at the preclinical and early clinical stages for various degenerative/fibrotic diseases. Indeed, MSCs have already demonstrated remarkable neuroregenerative and neuroprotective functional effects in several central nervous system disease models including clinical studies [11–13, 47]. The safe use of MSCs in autologous transplantations has led to several clinical trials for acute (ischemic stroke, spinal cord injury) or chronic (Parkinson’s disease) nervous system diseases. The eye is particularly attractive in that it has features which can rapidly advance cell therapy: the eye is readily accessible for injection and allows direct visualization and monitoring of the impact of therapeutic interventions. Moreover, little or no side effects may occur since local ocular administration of MSCs should not have the same general impact related to systemic injections. Lastly, in contrast to embryonic or fetal stem cells, the safety profile of MSCs and the possibility of obtaining MSCs from individual patients allowing autologous transplantation would highly facilitate transfer to the clinic. Despite all these advantages of MSCs therapy, many studies remain to be performed in order to define the cell-based therapy parameters for preclinical pharmacokinetics and toxicological studies—dose, biodistribution, safety evaluations—and also to identify some major molecules that could be involved in the positive effects observed in vitro and in vivo and that could be candidates for therapeutic development. Overall, present results show that MSCs are a promising tool for treating ocular hypertension and retinal cell degeneration. Such an innovative approach could therefore open many therapeutic avenues based on MSCs therapy in glaucoma.

## Additional file

Additional file 1: Figure S1. Showing MSCs characterization. Example of MSCs phenotyping in flow cytometry and microscopy immunofluorescence.

## Abbreviations

AC: Anterior chamber
AH: Aqueous humor
ATP: Adenosine triphosphate
BAC: Benzalkonium chloride
BDNF: Brain-derived neurotrophic factor
Brn3a: Brain-specific homeobox/POU domain protein 3A
BSA: Bovine serum albumin
CTG: CellTiter-Glo^®^
DAPI: 4′,6′-Diamidino-2-phénylindole
DMEM: Dulbecco’s modified Eagle’s medium
dMSCs: Differentiated mesenchymal stem cells
ECM: Extracellular matrix
ELISA: Enzyme-linked immunosorbent assay
EVC: Episcleral vein cauterization
FCS: Fetal calf serum
HPRT: Hypoxanthine guanine phosphoribosyltransferase
hTM: Human trabecular meshwork
IOP: Intraocular pressure
LC50: Lethal concentration 50
LE: Left eye
MEM: Minimal essential medium
MLC: Myosin
MSCs: Mesenchymal stem cells
MSC-CM: Conditioned medium of mesenchymal stem cells
NGF: Nerve growth factor
p-Akt: Phospho-Akt
PBS: Phosphate buffer solution
p-MLS: Phospho-myosin
POAG: Primary open-angle glaucoma
PS: Penicillin/streptomycin
QD: Quantum dot
RE: Right eye
RGCs: Retinal ganglion cells
TGF-β_2_: Transforming growth factor beta 2
TM: Trabecular meshwork
TMT: Trabecular mesenchymal transition
VEGF: Vascular endothelial growth factor
WB: Western blot
α-SMA: Alpha-smooth muscle actin
