# Supplementary material for: Intraocular pressure reduction and neuroprotection conferred by bone marrow-derived mesenchymal stem cells in an animal model of glaucoma
Source: Stem Cell Res Ther. 2015 Sep 16;6(1):177. doi: 10.1186/s13287-015-0168-0 (PMC4574127; doi:10.1186/s13287-015-0168-0)
Supplement: Additional file 1: Figure S1. — Showing MSCs characterization. Example of MSCs phenotyping in flow cytometry and microscopy immunofluorescence. [file 13287_2015_168_MOESM1_ESM.pdf]

Additional file 1: MSCs characterization

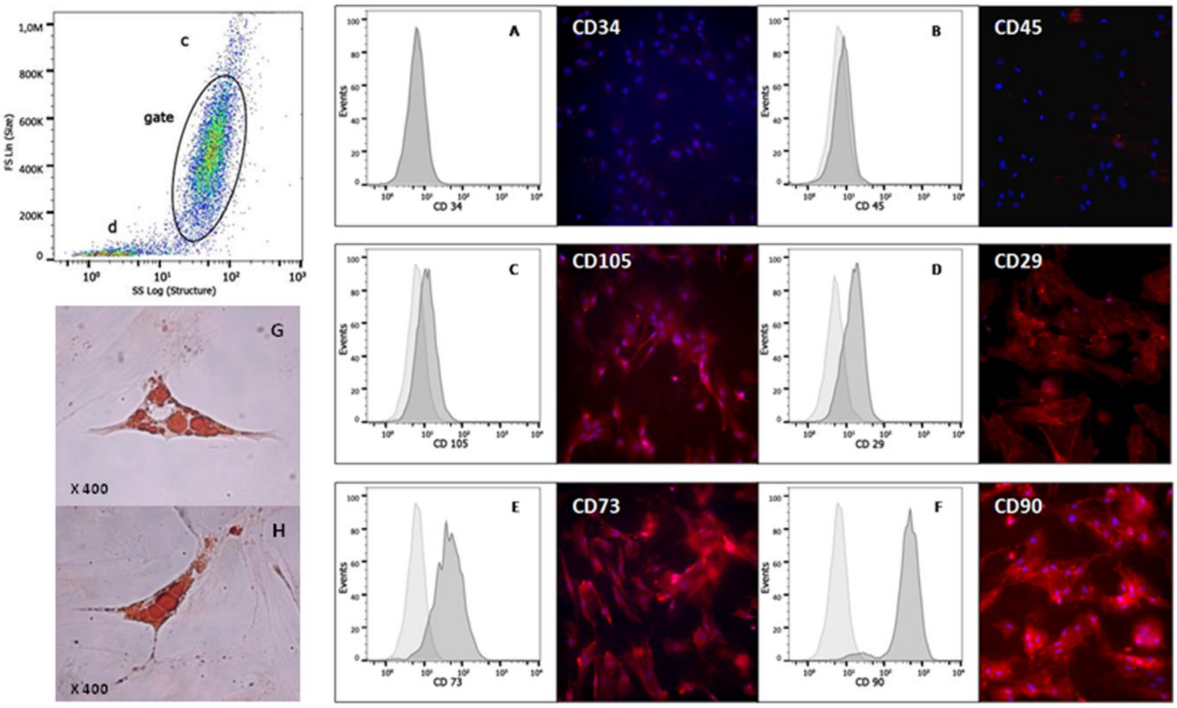

Example of MSCs phenotyping in flow cytometry and microscopy immunofluorescence. Upper left: Size (Forward Scatter, FS)/Structure (Side Scatter, SS) diagram showing the MSC population of interest (gate) excluding cell debris (d) and agreagates (c). A, B, C, D, E, and F: respective fluorescence histograms overlaid with their respective isotypic-matched immunoglobulin (negative control) of the positive markers CD73, CD90, CD105, CD29, and the negative markers CD34 and CD45. The following mouse anti-rat antibodies, CD45 (L-CA) and CD90 (Cedarlane, Burlington, Ontario), CD73 and CD29-PE (BD Pharmingen, San Jose, CA, USA), CD34 (Tebu-bio, Le Perray-en-Yvelines, France), were used for flow cytometry analyses (FC500® Beckman Coulter, Miami, CA, USA). An Alexa Fluor® 488 Goat Anti-Mouse immunoglobulin and an Alexa Fluor® 594 Goat Anti-Mouse immunoglobulin (Life Technologies; Carlsbad, CA, USA) were used as secondary

antibodies respectively for FCM and fluorescence microscopy (DM6000; Leica-microsystems; Nanterre, France).

MSCs were tested for adipogenic differentiation. Epifluorescence microscopy image of a MSCs stained with oil red (Sigma, Oakville, ON, Can) showing the presence of lipid vesicles confirming the MSCs adipogenic differentiation after 3 week-incubation in a differentiation medium: 10% FBS, 15 mmol/l hepes, 10 mol/l dexamethasone, 5 µg/ml insulin and 50 µg/ml indomethacin, were performed (G, H).
